# Different cardiovascular risks associated with elevated creatinine-based eGFR and cystatin C-based eGFR

**DOI:** 10.1038/s44325-024-00005-x

**Published:** 2024-05-02

**Authors:** Mengyi Liu, Ziliang Ye, Panpan He, Qimeng Wu, Sisi Yang, Yanjun Zhang, Chun Zhou, Yuanyuan Zhang, Fan Fan Hou, Xianhui Qin

**Affiliations:** Division of Nephrology, Nanfang Hospital, Southern Medical University, National Clinical Research Center for Kidney Disease, State Key Laboratory of Organ Failure Research, Guangdong Provincial Institute of Nephrology, Guangdong Provincial Key Laboratory of Renal Failure Research, 510515 Guangzhou, China

**Keywords:** Cardiovascular diseases, Stroke

## Abstract

To compare the association of elevated estimated glomerular filtration rate (eGFR) based on creatinine (eGFRcr) and cystatin C (eGFRcys) with the risk of cardiovascular diseases (CVD) and chronic kidney diseases (CKD). 372,060 participants free of CVD and CKD in the UK Biobank were included. Participants were categorized into low, normal and high eGFR groups according to the age- and sex-specific 5th and 95th percentiles of eGFR. The primary outcome was incident CVD, defined as a combination of ischemic heart disease, stroke, heart failure, and atrial fibrillation. Thresholds for high eGFR varied with age and sex, ranging from 96.5 to 116.0 mL/min/1.73 m^2^ and 100.3 to 120.1 mL/min/1.73 m^2^ for eGFRcr and eGFRcys, respectively. During a median follow-up of 12.4 years, 39,855 (10.7%) participants developed CVD. Compared with normal eGFR levels, high eGFRcr levels were associated with a higher risk of CVD (HR, 1.19; 95% CI: 1.14–1.25), while high eGFRcys levels were associated with a lower risk of CVD (HR, 0.90; 95% CI: 0.85–0.95). Compared to normal eGFR levels, both high eGFRcr and high eGFRcys levels were related to a lower risk of CKD. Elevated eGFRcr levels were associated with a higher risk of CVD, and elevated eGFRcys levels were associated with a lower risk of CVD.

## Introduction

Chronic kidney disease (CKD) is a global health and socioeconomic burden, with an estimated global prevalence of 9.1%^1^. CKD may increase the risk of end-stage kidney disease (ESKD) and cardiovascular diseases (CVD)^2^. Some^3–9^ previous studies^3–10^ have shown that abnormally elevated glomerular filtration rate (GFR) may also confer a higher risk of CVD. However, the impact of abnormally elevated GFR on CVD risk has not been fully evaluated and is often overlooked. It is difficult to draw accurate conclusions based on previous research data for the following reasons:

First, most previous studies have simply used a variety of arbitrarily absolute values of estimated GFR (eGFR) to define abnormally elevated eGFR without considering sex-based differences and age-related physiological declines in GFR^11^. Second, almost all previous studies estimated GFR only by serum creatinine. One potential pitfall is that the increased risk of CVD associated with elevated GFR levels in these studies could simply be due to muscle wasting resulting in reduced creatinine concentrations, leading to an overestimation of GFR levels^12^. Serum cystatin C is less sensitive to differences in muscle mass than serum creatinine and may be a better filtration marker. Especially at higher levels of GFR, the association between cystatin C and the risk of mortality is stronger and more linear than serum creatinine^12–14^. However, there is limited evidence of an association between cystatin C-based eGFR (eGFRcys) elevation and the risk of CVD in healthy individuals. To date, only one cohort has assessed the relationship of elevated eGFRcys with the risk of coronary heart disease (CHD) and heart failure (HF), but no significant associations were observed^15^. Therefore, the effect of elevated eGFRcys on CVD risk warrants further investigation.

As such, we aimed to investigate the relationship between elevated eGFR, including eGFRcys and eGFR based on serum creatinine (eGFRcr), and the risk of incident CVD and its relatively common subtypes (including ischemic heart disease [IHD], stroke, HF, and atrial fibrillation [AF])^16,17^.

## Results

### Baseline population characteristics

Ultimately, 372,060 participants were enrolled in the present study (Fig. S1). The mean (standard deviation [SD]) age was 55.7 (8.0) years, and 206,483 (55.5%) were female. The distribution of eGFR for each age category by sex is shown in Table S1. Thresholds for high eGFR varied with age. eGFRcr ranged from 96.5 to 115.4 mL/min/1.73 m^2^ for female participants and 96.7 to 116.0 mL/min/1.73 m^2^ for male participants, respectively, while eGFRcys ranged from 100.3 to 118.1 mL/min/1.73 m^2^ for female participants and 104.5–120.1 mL/min/1.73 m^2^ for male participants, respectively.

As shown in Table 1, compared to normal eGFRcys, participants with high eGFRcys tended to be younger, non-white race, non-smokers, and had lower levels of body mass index (BMI), blood pressure (BP), triglycerides (TG), total cholesterol (TC), and high-sensitivity C reactive protein (hs-CRP), and higher prevalence of diabetes. However, compared with participants with normal eGFRcr, those with high eGFRcr tended to be smokers and to be taking antihypertensive and cholesterol-lowering drugs and had higher levels of BMI, BP, and TG, and higher prevalence of diabetes (Table 1). Moreover, participants with higher age- and sex-specific deciles of eGFRcr were more likely to be smokers and had higher systolic BP (SBP) levels and higher prevalence of diabetes, while participants with higher deciles of eGFRcyc were younger and more likely to be non-smokers, and had lower levels of BMI, BP, TG and hs-CRP (Tables S2 and S3). In addition, compared with participants with high eGFRcys and high eGFRcr, those with high eGFRcr but low or normal eGFRcys tended to be smokers and to be taking the antihypertensive drug and had higher BMI, TG, and hs-CRP levels (Table S4).

**Table 1:** Baseline population characteristics by eGFR categories^a^

	eGFRcr					eGFRcyc			
	Low	Normal	High	*P* value		Low	Normal	High	*P* value
*N*	18,575	334,919	18,566			18,573	334,932	18,555	
Age, years	55.9 (8.0)	55.7 (8.0)	55.2 (8.0)	<0.001		55.9 (8.0)	55.7 (8.0)	55.3 (8.0)	<0.001
Male, No. (%)	8265 (44.5)	149,049 (44.5)	8263 (44.5)	0.999		8266 (44.5)	149,055 (44.5)	8256 (44.5)	0.999
White, No. (%)	17,709 (95.3)	320,168 (95.6)	15,533 (83.7)	<0.001		17,480 (94.1)	318,573 (95.1)	17,357 (93.5)	<0.001
Body mass index, kg/m^2^	27.7 (4.3)	27.1 (4.5)	27.2 (5.3)	<0.001		30.4 (5.9)	27.1 (4.4)	25.2 (3.5)	<0.001
Systolic blood pressure, mmHg	136.6 (18.0)	137.1 (18.2)	138.3 (18.5)	<0.001		138.4 (18.1)	137.1 (18.2)	136.4 (18.5)	<0.001
Diastolic blood pressure, mmHg	82.3 (10.0)	82.2 (9.9)	82.5 (10.1)	<0.001		84.2 (10.2)	82.2 (9.9)	80.9 (9.8)	<0.001
Smoking status, No. (%)				<0.001					<0.001
Never	11,097 (59.7)	189,458 (56.6)	9460 (51.0)			9110 (49.0)	190,085 (56.8)	10,820 (58.3)	
Former	6189 (33.3)	112,000 (33.4)	5927 (31.9)			5530 (29.8)	111,832 (33.4)	6754 (36.4)	
Current	1232 (6.6)	32,421 (9.7)	3112 (16.8)			3850 (20.7)	31,977 (9.5)	938 (5.1)	
Antihypertensive drug use, No. (%)	3419 (18.5)	50,138 (15.1)	3626 (19.8)	<0.001		4582 (24.9)	50,195 (15.1)	2406 (13.0)	<0.001
Cholesterol-lowering drug use, No. (%)	2514 (13.6)	38,117 (11.4)	2585 (14.1)	<0.001		2836 (15.4)	38,196 (11.5)	2184 (11.8)	<0.001
History of diabetes, No. (%)	557 (3.2)	12,629 (4.0)	1890 (10.8)	<0.001		1187 (6.7)	12,937 (4.1)	952 (5.4)	<0.001
Total cholesterol, mmol/L	5.7 (1.1)	5.8 (1.1)	5.7 (1.2)	<0.001		5.7 (1.2)	5.8 (1.1)	5.7 (1.1)	<0.001
High-density lipoprotein cholesterol, mmol/L	1.4 (0.4)	1.5 (0.4)	1.5 (0.4)	<0.001		1.3 (0.3)	1.5 (0.4)	1.6 (0.4)	<0.001
Triglycerides, mmol/L	1.8 (1.0)	1.7 (1.0)	1.8 (1.2)	<0.001		2.1 (1.1)	1.7 (1.0)	1.4 (0.9)	<0.001
C-reactive protein, mg/L	2.4 (3.6)	2.4 (3.9)	2.9 (4.8)	<0.001		4.1 (5.4)	2.3 (3.9)	1.6 (3.2)	<0.001
Creatinine, mg/dL	1.0 (0.1)	0.8 (0.1)	0.6 (0.1)	<0.001		0.9 (0.2)	0.8 (0.1)	0.7 (0.1)	<0.001
Cystatin C, mg/L	1.0 (0.1)	0.9 (0.1)	0.8 (0.1)	<0.001		1.1 (0.1)	0.9 (0.1)	0.7 (0.1)	<0.001
eGFRcr, mL/min/1.73 m^2^	68.1 (4.7)	92.4 (9.9)	108.3 (7.6)	<0.001		83.7 (12.4)	92 (11.3)	99.7 (9.7)	<0.001
eGFRcyc, mL/min/1.73 m^2^	82.1 (13.7)	91.8 (13.9)	101.3 (12.4)	<0.001		66.9 (5.4)	92.0 (12.6)	113.0 (7.0)	<0.001

^a^Values are presented as means (SD) or proportions.

*eGFR* estimated glomerular filtration rate, *eGFRcr* eGFR based on creatinine, *eGFRcys* eGFR based on cystatin C.

### Relationship of eGFR categories with incident CVD

During a median follow-up of 12.4 years (4,393,086 person-years), 39,855 (10.7%) participants developed CVD, while 21,5596 (5.8%), 7496 (2.0%), 6341 (1.7%), 166,107 (4.3%) participants developed IHD, stroke, HF and AF, respectively.

In multivariable Cox models, compared to normal eGFRcr, participants with high eGFRcr had higher risks of incident CVD (HR, 1.19; 95% CI: 1.14–1.25), IHD (HR, 1.11; 95% CI: 1.04–1.18), stroke (HR, 1.46; 95% CI: 1.32–1.61), HF (HR, 1.50; 95% CI: 1.35–1.66) and AF (HR, 1.20; 95% CI: 1.11–1.29) (Table 2). Further adjusting for BMI did not materially change the magnitude and the significance. Nevertheless, eGFRcys showed an inverse relationship with the risk of incident CVD, with a higher risk of incident CVD in the low eGFRcys group (HR, 1.32; 95% CI: 1.27–1.37) and a lower risk of incident CVD in high eGFRcys group (HR, 0.90; 95% CI: 0.85–0.95), compared to normal eGFRcys group (Table 2). Similar findings were obtained for incident IHD, stroke, HF and AF, respectively (Table 2). Additionally, further adjusting for BMI resulted in modestly attenuation of the risk of incident CVD associated with high eGFRcys (Table 2).

**Table 2:** The relationship of eGFR categories with risk of incident adverse cardiovascular events

	eGFRcr				eGFRcys		
	Low eGFR	Normal eGFR	High eGFR		Low eGFR	Normal eGFR	High eGFR
*Cardiovascular disease*							
Events (Incidence rates^a^)	1970(9.1)	35,498(9.0)	2387(11.0)		2893(13.7)	35,318(8.9)	1644(7.4)
Crude model	1.03(0.98, 1.08)	ref	1.23(1.18, 1.29)		1.56(1.50, 1.62)	ref	0.83(0.79, 0.87)
Adjusted model 1^b^	1.02(0.98, 1.08)	ref	1.19(1.14, 1.25)		1.36(1.30, 1.42)	ref	0.90(0.85, 0.95)
Adjusted model 2^b^	1.01(0.97, 1.07)	ref	1.21(1.15, 1.26)		1.29(1.24, 1.35)	ref	0.93(0.88, 0.98)
*Ischemic heart disease*							
Events (Incidence rates^a^)	1103(5.0)	19,250(4.8)	1243(5.6)		1645(7.5)	19,078(4.7)	873(3.9)
Crude model	1.04(0.98, 1.10)	ref	1.18(1.11, 1.25)		1.60(1.53, 1.69)	ref	0.82(0.77, 0.88)
Adjusted model 1^b^	1.03(0.97, 1.10)	ref	1.11(1.04, 1.18)		1.32(1.25, 1.40)	ref	0.92(0.86, 0.99)
Adjusted model 2^b^	1.02(0.96, 1.09)	ref	1.11(1.05, 1.19)		1.28(1.21, 1.35)	ref	0.94(0.88, 1.02)
*Stroke*							
Events (Incidence rates^a^)	353(1.6)	6603(1.6)	540(2.4)		556(2.5)	6603(1.6)	337(1.5)
Crude model	0.97(0.87, 1.08)	ref	1.50(1.37, 1.64)		1.56(1.43, 1.70)	ref	0.92(0.82, 1.03)
Adjusted model 1^b^	0.97(0.86, 1.09)	ref	1.46(1.32, 1.61)		1.42(1.29, 1.56)	ref	0.94(0.83, 1.06)
Adjusted model 2^b^	0.97(0.86, 1.09)	ref	1.45(1.32, 1.60)		1.43(1.30, 1.58)	ref	0.93(0.83, 1.05)
*Heart failure*							
Events (Incidence rates^a^)	304(1.3)	5538(1.4)	499(2.2)		624(2.8)	5488(1.3)	229(1.0)
Crude model	1.00(0.89, 1.12)	ref	1.65(1.51, 1.81)		2.11(1.94, 2.29)	ref	0.75(0.66, 0.86)
Adjusted model 1^b^	0.99(0.87, 1.12)	ref	1.50(1.35, 1.66)		1.71(1.56, 1.87)	ref	0.79(0.69, 0.92)
Adjusted model 2^b^	0.97(0.86, 1.10)	ref	1.52(1.37, 1.68)		1.51(1.38, 1.66)	ref	0.86(0.75, 1.00)
*Atrial fibrillation*							
Events (Incidence rates^a^)	782(3.5)	14,385(3.5)	940(4.2)		1123(5.1)	14,317(3.5)	667(3.0)
Crude model	0.99(0.92, 1.06)	ref	1.20(1.12, 1.28)		1.45(1.37, 1.54)	ref	0.84(0.77, 0.90)
Adjusted model 1^b^	0.96(0.89, 1.04)	ref	1.20(1.11, 1.29)		1.33(1.25, 1.42)	ref	0.88(0.80, 0.95)
Adjusted model 2^b^	0.95(0.88, 1.02)	ref	1.22(1.14, 1.32)		1.20(1.12, 1.28)	ref	0.94(0.86, 1.02)

^a^Incidence rates per 1000 person-years.

^b^Adjusted Model 1: Adjusted for age, sex, race, systolic blood pressure, diastolic blood pressure, smoking status, history of diabetes, antihypertensive drug use, cholesterol-lowering drug use, triglycerides, total cholesterol, high-density lipoprotein cholesterol, and high-sensitivity C reactive protein; Adjusted Model 2: adjusted for the covariates in Model 1 and further adjusted for body mass index.

*eGFR* estimated glomerular filtration rate, *eGFRcr* eGFR based on creatinine, *eGFRcys* eGFR based on cystatin C.

Of note, compared with both normal eGFRcr and normal eGFRcys, both high eGFRcr and high eGFRcys were not significantly associated with subsequent risk of developing CVD, while high eGFRcr but low eGFRcys or high eGFRcr but normal eGFRcys was significantly related to a higher risk of incident CVD (Table S5). In addition, compared to a difference between eGFRcr and eGFRcys within ±15%, a difference of >15% was associated with a higher risk of incident CVD (HR, 1.23; 95% CI: 1.220–1.27), which was more obvious among those with high eGFRcr levels. Of note, a large difference doesn’t exist in the high eGFRcyc group (Table S6).

**Fig. 1:**
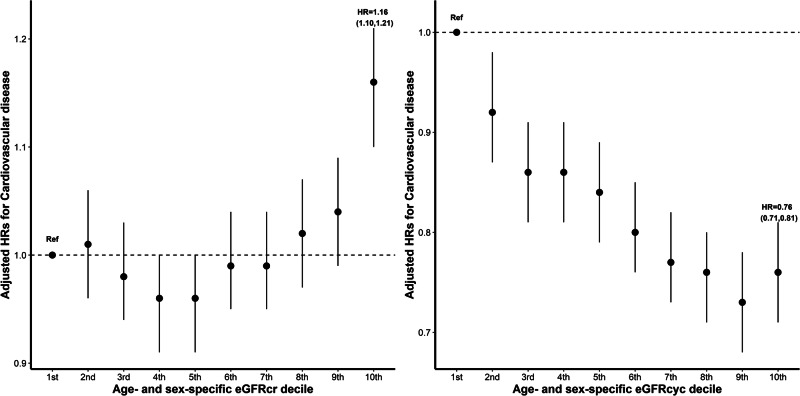
Adjusted hazard ratios for cardiovascular outcomes by age- and sex-specific eGFR deciles^*^. *Adjusted for age, sex, body mass index, race, systolic blood pressure, diastolic blood pressure, smoking status, history of diabetes, antihypertensive drug use, cholesterol-lowering drug use, triglycerides, total cholesterol, high-density lipoprotein cholesterol, and high-sensitivity C reactive protein. eGFR estimated glomerular filtration rate, eGFRcr eGFR based on creatinine, eGFRcys eGFR based on cystatin C.

To better describe the dose–response relationship of eGFR with cardiovascular outcomes, we further classified the study participants according to age- and sex-specific eGFR deciles. Using the lowest decile as a reference, participants in the highest eGFRcr decile were related to a significantly higher risk of incident CVD and each subtype, while participants in the highest eGFRcys decile exhibited significantly decreased risks of incident CVD and each subtype (Figs. 1 and S2).

### Relationship of eGFR categories with incident CKD

6621 (1.8%) participants had CKD during a median follow-up of 12.5 years. Overall, high eGFR showed an inverse relationship with the risk of incident CKD (Table S7), with a higher CKD risk in low eGFRcr (HR, 5.06; 95% CI: 4.74–5.39) or low eGFRcys (HR, 3.16; 95% CI: 2.94–3.40) group, and a lower CKD risk in high eGFRcr (HR, 0.38; 95% CI: 0.31–0.46) or high eGFRcys (HR, 0.33; 95% CI: 0.27–0.42) group, compared to corresponding normal eGFR group.

### Relationship of creatinine and cystatin C categories with incident CVD

According to the 5th and 95th percentiles of the age- and sex-specific creatinine and cystatin C, participants were classified into three groups: low, normal and high. Compared to normal creatinine, participants with low creatinine had higher risks of incident CVD (HR, 1.21; 95% CI: 1.15–1.26; Table 3). However, compared to those with normal cystatin C, participants with low cystatin C exhibited lower risks of incident CVD (HR, 0.91; 95% CI: 0.86–0.96; Table 3), and those with high cystatin C exhibited higher risks of incident CVD (HR, 1.30; 95% CI: 1.25–1.35; Table 3). Similar findings were obtained for incident IHD, stroke, HF and AF, respectively (Table 3).

**Table 3:** The relationship of creatinine and cystatin C categories with risk of incident adverse cardiovascular events

	Creatinine				Cystatin C		
	Low	Normal	High		Low	Normal	High
*Cardiovascular disease*							
Events (Incidence rates^a^)	2483(11.5)	35,455(8.9)	1917(8.9)		1633(7.5)	35,357(8.9)	2865(13.7)
Crude model	1.29(1.24, 1.35)	ref	1.01(0.96, 1.06)		0.84(0.80, 0.88)	ref	1.56(1.50, 1.62)
Adjusted model 1^b^	1.19(1.14, 1.25)	ref	1.02(0.97, 1.07)		0.88(0.84, 0.93)	ref	1.37(1.31, 1.42)
Adjusted model 2^b^	1.21(1.15, 1.26)	ref	1.01(0.96, 1.06)		0.91(0.86, 0.96)	ref	1.30(1.25, 1.35)
*Ischemic heart disease*							
Events (Incidence rates^a^)	1277(5.8)	19,252(4.8)	1067(4.8)		854(3.9)	19,122(4.7)	1620(7.5)
Crude model	1.22(1.15, 1.29)	ref	1.01(0.95, 1.08)		0.81(0.76, 0.87)	ref	1.59(1.51, 1.67)
Adjusted model 1^b^	1.10(1.03, 1.17)	ref	1.02(0.95, 1.09)		0.90(0.84, 0.97)	ref	1.31(1.24, 1.39)
Adjusted model 2^b^	1.11(1.04, 1.18)	ref	1.01(0.95, 1.08)		0.92(0.85, 0.99)	ref	1.27(1.20, 1.35)
*Stroke*							
Events (Incidence rates^a^)	534(2.4)	6608(1.6)	354(1.6)		339(1.5)	6615(1.6)	542(2.4)
Crude model	1.48(1.36, 1.62)	ref	0.98(0.88, 1.09)		0.94(0.84, 1.05)	ref	1.53(1.40, 1.67)
Adjusted model 1^b^	1.43(1.30, 1.57)	ref	0.99(0.88, 1.11)		0.94(0.83, 1.06)	ref	1.41(1.28, 1.56)
Adjusted model 2^b^	1.43(1.30, 1.57)	ref	0.99(0.88, 1.11)		0.93(0.82, 1.05)	ref	1.43(1.29, 1.57)
*Heart failure*							
Events (Incidence rates^a^)	532(2.4)	5510(1.3)	299(1.3)		225(1.0)	5500(1.3)	616(2.8)
Crude model	1.77(1.62, 1.94)	ref	0.99(0.88, 1.12)		0.75(0.65, 0.85)	ref	2.09(1.93, 2.27)
Adjusted model 1^b^	1.52(1.38, 1.68)	ref	0.99(0.87, 1.12)		0.75(0.65, 0.87)	ref	1.71(1.56, 1.87)
Adjusted model 2^b^	1.55(1.40, 1.71)	ref	0.98(0.86, 1.11)		0.81(0.70, 0.94)	ref	1.51(1.37, 1.66)
*Atrial fibrillation*							
Events (Incidence rates^a^)	1010(4.6)	14,340(3.5)	757(3.4)		666(3.0)	14,323(3.5)	1118(5.1)
Crude model	1.30(1.22, 1.38)	ref	0.96(0.90, 1.04)		0.85(0.79, 0.92)	ref	1.46(1.37, 1.55)
Adjusted model 1^b^	1.20(1.12, 1.29)	ref	0.97(0.89, 1.05)		0.86(0.79, 0.93)	ref	1.36(1.27, 1.45)
Adjusted model 2^b^	1.23(1.15, 1.32)	ref	0.95(0.88, 1.03)		0.92(0.84, 1.00)	ref	1.22(1.14, 1.31)

^a^Incidence rates per 1000 person-years.

^b^Adjusted Model 1: Adjusted for age, sex, race, systolic blood pressure, diastolic blood pressure, smoking status, history of diabetes, antihypertensive drug use, cholesterol-lowering drug use, triglycerides, total cholesterol, high-density lipoprotein cholesterol, and high-sensitivity C reactive protein; Adjusted Model 2: adjusted for the covariates in Model 1 and further adjusted for body mass index.

### Stratified analyses and sensitivity analyses

**Fig. 2:**
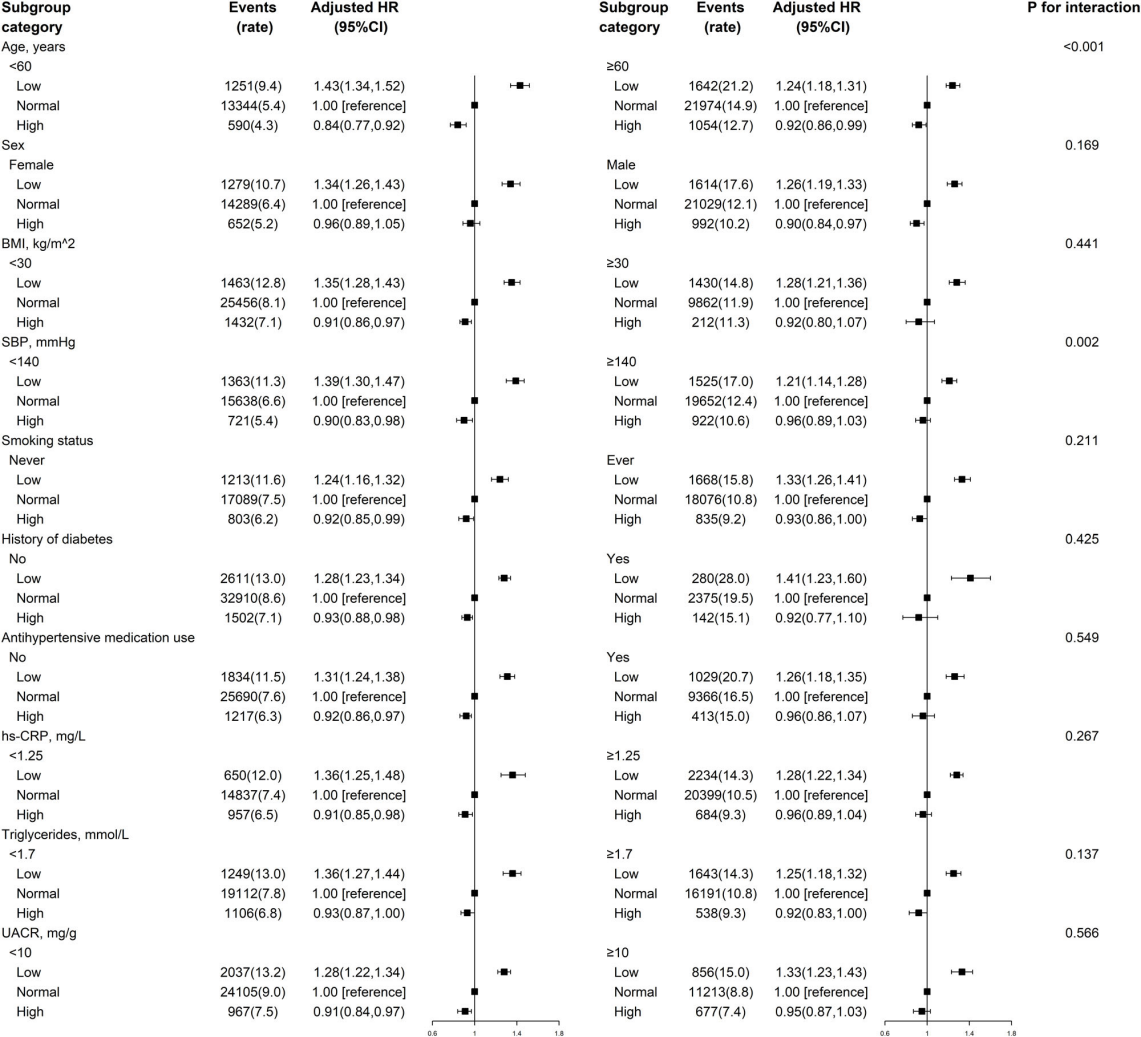
The association between eGFRcys categories and risk of incident CVD in various subgroups^*^. *Adjusted for age, sex, body mass index, race, systolic blood pressure, diastolic blood pressure, smoking status, history of diabetes, antihypertensive drug use, cholesterol-lowering drug use, triglycerides, total cholesterol, high-density lipoprotein cholesterol, and high-sensitivity C reactive protein, if not already stratified. BMI body mass index, CVD cardiovascular disease, eGFR estimated glomerular filtration rate, eGFRcys eGFR based on cystatin C, hs-CRP high-sensitivity C reactive protein, SBP systolic blood pressure, UACR urine albumin:creatinine ratio.

In stratified analyses, the eGFRcys and eGFRcr categories showed similar trends in association with CVD risk across different subgroups of age, sex, BMI, history of diabetes, antihypertensive drug use, hs-CRP, TG, and urine albumin: creatinine ratio (UACR) levels, although some interactions had *P*-values lower than 0.05 (Figs. 2, S3, and Table S8).

In sensitivity analyses, the main findings remained robust (Table S9). Firstly, when eGFR was explored as a continuous variable or quartiles, eGFRcr was positively associated with the risk of incident CVD (per SD increment, HR, 1.03; 95% CI: 1.02–1.05), whereas eGFRcys was inversely associated with the risk of incident CVD (per SD increment, HR, 0.89; 95% CI: 0.88–0.90). Similarly, when eGFR was categorized according to clinical definition, compared with eGFR at 90-105 mL/min/1.73 m^2^, eGFRcr at 105–120 mL/min/1.73 m^2^ was related to a higher risk of incident CVD (per SD increment, HR, 1.16; 95% CI: 1.10–1.22), while eGFRcys at 105–120 mL/min/1.73 m^2^ was related to a lower risk of incident CVD (per SD increment, HR, 0.91; 95% CI: 0.88–0.95). Secondly, similar results were observed when Cox models were stratified by age and sex, or performed without adjustments for age and sex, or when the competing risk of death was taken into account.

## Discussion

In this large cohort of participants without prior CVD or CKD, we demonstrated that high eGFRcys was related to lower risk of incident CVD and its each subtype, while high eGFRcr was related to higher risk of incident CVD and its each subtype. Furthermore, both high eGFRcr and high eGFRcys were related to a lower risk of CKD incidence.

Though most previous studies suggested that elevated eGFR was positively related to CVD incidence in general population^3–5^ or populations at high-risk conditions^6–9^, these studies have used a variety of arbitrary absolute eGFR cut-off values, and did not consider sex differences and age-related physiological decline in eGFR^11^, which made the conclusions of these studies unreliable and difficult to compare with one another. In fact, only a few data were available on the association of elevated eGFR according to age- and sex-matched eGFRcr threshold with the risk of CVD. Park et al.^4^ found that elevated eGFR adjusted for age, sex, muscle mass, and history of diabetes and/or hypertension medication was related to higher risk of developing CVD in 23,824 apparently healthy Korean adults. Using a comparable definition of elevated eGFR, higher risk of CVD related to elevated eGFR was also observed in the ABP-International study including predominantly hypertensive individuals^6^ and the CARTaGENE populational cohort including healthy middle-aged individuals^3^. Consistently, based on a large-scale population-based study of British general populations, our current study determined the similar associations of high eGFRcr with incident CVD and its subtypes, and observed the strongest association for HF.

Nevertheless, based on age- and sex-specific definition, our analyses from the long-term follow-up of general population demonstrated that high eGFRcys was related to lower risk of incident CVD and its subtypes. Consistently, Waheed et al.^15^, observed a stronger and more linear inverse associations of eGFRcys with both CHD and HF compared with eGFRcr, but did not find a significant relationship of elevated eGFRcys with CHD and HF. Indeed, although higher eGFR was considered be detrimental to the kidney through changes in the hemodynamics of glomerular capillaries accompanied by glomerular hypertrophy and elevated glomerular pressure in obese and diabetic individuals^18–20^, elevated GFR without glomerular hypertension (i.e., simultaneous increase in GFR and renal plasma flow with normal filtration fraction) does not result in damage in glomerulus^18^. In our study, both high eGFRcr and eGFRcys were related to lower risk of CKD incidence, further suggesting that a physiological state with high eGFR but no glomerular hypertension in an apparently healthy population may not have adverse effects.

The possible reasons for the difference in cardiovascular risk between elevated eGFRcr and eGFRcys is the non-GFR determinant of serum creatinine and cystatin C, considering that we did observe in the current study that low serum creatinine level was associated with a higher risk of CKD, while low cystatin C level was associated with a lower risk of CKD. It has been reported that non-GFR determinants of serum creatinine (such as muscle mass, diet, and physical activity) may be confounding factors in the relationship between eGFRcr and disease endpoints^14,21^, and cystatin C is considered to be a better marker of kidney function because it is less sensitive to non-GFR determinants than creatinine^22^. Compared to eGFRcr, eGFRcys may provide a more valid estimate of GFR especially at high GFR levels^14,15^. Accordingly, we observed that both high eGFRcr and high eGFRcys were not significantly associated with CVD risk, and that the higher risk of CVD associated with high eGFRcr was primarily driven by high eGFRcr and low or normal eGFRcys. Of note, among those with higher eGFRcr levels, those with lower eGFRcys levels were more likely to be smokers, tended to be unhealthy, and had higher levels of BMI, TG and hs-CRP and had a higher prevalence of diabetes and use of antihypertensive medications. Nevertheless, we controlled for these potential confounders in the multivariable models and did not observe substantial differences in the stratified analyses, suggesting that these factors do not fully explain our findings.

Another possible interpretation is that a high eGFRcr but a low or normal eGFRcys suggests the presence of ‘shrunken pore syndrome’ (SPS)^23^. Since cystatin C (13.3 kDa) is >100 times larger than creatinine (113 Da), cystatin C can no longer be excreted from the bloodstream thought glomerular pores as the glomerular pores shrink, resulting in high eGFRcr but low eGFRcys. SPS may lead to an increase in serum atherosclerosis-promoting proteins and middle-molecular-weight proteins, which are associated with an increased risk of CVD^24,25^. Consistently, previous studies have observed that significantly higher eGFRcr than eGFRcys was associated with a higher risk of CVD^25–27^. Our sensitivity analysis also found that significantly higher eGFRcr than eGFRcys was associated with a higher risk of CVD, with a large difference between eGFRcr and eGFRcys occurring primarily in the group with a high eGFRcr but not in the group with a high eGFRcyc. These findings further suggest that SPS may partially explain the different cardiovascular risks associated with high eGFRcr and eGFRcy.

To our knowledge, the study is the largest one to compare the effect of high eGFRcr and eGFRcys on developing CVD and CKD. Nevertheless, this work has some limitations. First, eGFR was calculated using a formula instead of direct measurements. Nevertheless, the CKD-EPI equation^28^ is a standard method for estimating GFR, particularly in an epidemiologic setting. Second, eGFR was calculated based on a one-time measurement of serum cystatin C and creatinine at baseline, and the magnitude of decline in eGFRcr or eGFRcys in relation to CVD was not assessed in the current study. Third, given that the distribution of eGFR varies widely across racial/ethnic groups, the small proportion of non-Caucasians in the UK Biobank may lead to limited generalization of the results to other racial/ethnic groups. Moreover, the UK Biobank participants were recruited over a 30-year range of ages, and healthier than the general UK population^29^, and those with prevalent CVD were excluded in the current study, all of which may lead to a possible underestimation of associations. Fourth, the strategy of covariates selection was only based on background knowledge, which may produce bias. However, the stepwise regression analysis was used to select covariates, and the results also showed that the model containing all the aforementioned covariates has the smallest Akaike information criterion (AIC)^30^. Moreover, when variance inflation factors (VIFs) are >5, multicollinearity is considered high, and no significant multicollinearity was found in the current study.

In summary, data from UK Biobank demonstrated that elevated eGFRcr was related to higher risks of cardiovascular outcomes, while high eGFRcys was related to lower CVD hazards in healthy participants without CVD and CKD at baseline. Moreover, both high eGFRcr and high eGFRcys were related to a lower risk of incident CKD. Non-GFR determining factors of creatinine and cystatin C or the presence of SPS may probably explain the different cardiovascular risks associated with high eGFRcr and eGFRcys.

## Methods

### Study design and population

The current study was conducted based on the UK Biobank, a large population-based cohort recruiting about 500,000 adults in the United Kingdom between 2006 and 2010. As described previously^31,32^, participants were asked to complete touchscreen questionnaires, face-to-face interviews and a variety of physical measurements, along with provide biological samples. The UK Biobank was approved by the North West Research Ethics Committee, and all participants signed informed consent forms.

In the current study, we included participants with completed information on kidney function and CVD and without prior CVD or CKD. Of the 380,527 participants, those with a BMI of <18.5 kg/m^2^ (*n* = 2845) or with a follow-up period of <2 years (*n* = 5622) were further excluded to reduce the potential effects of reverse causality, leading to the final 372,060 participants included (Fig. S1).

### Measurements of variables

The procedures for the collection and handling of biological samples have been described and validated previously^33^. Biochemistry measures were performed at a dedicated central laboratory, including creatinine, cystatin C, lipids (TG, TC, high-density lipoprotein cholesterol [HDL-C]), hs-CRP, and urine albumin content.

Detailed information on covariates was available through standardized questionnaires. BP measurements were performed manually or automatedly, and the average value of the two BP measurements was used. Prevalent diabetes at baseline was distinguished by multiple procedures taking diabetes type and diagnosis sources into account^34^, and history of diabetes was defined as prevalent diabetes, or hemoglobinA1c (HbA1c) ≥ 6.5%.

### Definitions of low, normal, and high glomerular filtration

eGFRcr and eGFRcys were calculated by the CKD-EPI equation based on serum creatinine or cystatin C separately^28^. Participants were categorized into three groups: low, normal and high eGFR, according to the age- and sex-specific 5th and 95th percentiles of eGFR, that is the 5th and 95th percentiles of each eGFR subgroup categorized by age (<45, 45–50, 50–55, 55–60, 60–65, and ≥65 years) and sex (females and males) (Table S1).

### Ascertainment of outcomes

The incidence of outcomes was mainly determined through the use of linkage with hospital admission data and death register data based on the International Classification of Diseases (ICD) edition 9, ICD edition 10, and the Office of Population Censuses and Surveys Classification of Interventions and Procedures, version 4 (OPCS-4)

The primary outcome in the present study was CVD incidence, defined as a combination of incident IHD, stroke, HF, and AF, using the corresponding code in Table S2. The secondary outcomes were incident IHD, stroke, HF and AF, separately; and incident CKD (Table S10).

### Statistical analysis

Comparisons of characteristics based on eGFR categories were evaluated though *χ*^2^ tests and ANOVA tests for categorical and continuous variables, respectively.

With normal eGFR as the reference group, Cox proportional hazards models were conducted using R package *survival* to assess the relationship of eGFR categories with cardiovascular outcomes. Potential covariates that were known to be traditional or suspected risk factors for kidney diseases and CVD were adjusted for, including age, sex, BMI, race, SBP, diastolic BP (DBP), smoking status, history of diabetes, antihypertensive drug use, cholesterol-lowering drug use, TC, HDL-C, TG and hs-CRP. Proportional hazard assumptions for Cox regress models were verified by the interaction between exposures and log-transformed follow-up time, and no clear evidence of violation was detected.

To further assess the dose–response relationship of eGFR with study outcomes, we divided participants into narrower groups according to the age- and sex-specific deciles of eGFR (i.e. participants were first classified by age [<45, 45–50, 50–55, 55–60, 60–65, and ≥65 years] and sex categories [females and males], with each subgroup further divided into ten groups based on deciles of eGFR in each subgroup), and the lowest decile was used as the reference group to estimate the relationship between eGFR decile and study outcomes. In addition, we further determined the association of eGFR categories with CKD incidence to assess whether the relation of elevated eGFR with CVD was related to early stages of renal dysfunction. Moreover, we also determined the association of age- and sex-specific creatinine and cystatin C categories with incident CVD.

To investigate the potential modifiers on the relation of eGFR categories with CVD, stratified analyses were further assessed and interactions between subgroups and eGFR categories were evaluated by likelihood ratio testing.

Several sensitivity analyses were assessed. First, eGFR was investigated as a continuous variable and categorical variable according to quartiles and clinical definition. Second, since age and sex are part of the eGFR formulas, we further performed Cox models stratified by age and sex, or Cox models without adjustments for age and sex. Third, because death is a competing risk for CVD incidence, a Fine-Gray competing risk model was performed using the R package *cmprsk* to investigate the relationship of eGFR categories with cardiovascular outcomes while setting mortality as a competing risk.

A two-tailed *P* < 0.05 was considered to be statistically significant in all analyses. All statistical analyses were performed using R 4.1.1 software (http://www.R-project.org/).

## Supplementary information

Supplementary Information

## Data Availability

The UK Biobank data are available on application to the UK Biobank.

## Code availability

The analytic methods, code, and study materials that support the findings of this study will be available from the corresponding authors on request.
