# Supplementary material for: Different cardiovascular risks associated with elevated creatinine-based eGFR and cystatin C-based eGFR
Source: NPJ Cardiovasc Health. 2024 May 2;1:3. doi: 10.1038/s44325-024-00005-x (PMC12912296; doi:10.1038/s44325-024-00005-x)
Supplement: Supplementary file 1 — Supplementary Information [file 44325_2024_5_MOESM1_ESM.pdf]

Supplementary Online Content

Figure S1. Flow chart of the participants in the current analysis.

Figure S2. Adjusted hazard ratios for second cardiovascular outcomes by age- and sex-specific eGFR deciles.

Figure S3. The association between eGFRcr categories and risk of incident CVD in various subgroups.

Table S1. Distribution of eGFR by sex and age.

Table S2. Baseline population characteristics by the age- and sex-specific deciles of eGFRcr.

Table S3. Baseline population characteristics by the age- and sex-specific deciles of eGFRcyc.

Table S4. Baseline population characteristics by eGFRcr and eGFRcys categories.

Table S5. Combined association of eGFRcr categories and eGFRcyc categories with CVD.

Table S6. Association of difference between eGFRcr and eGFRcys (eGFRcys minus eGFRcr divided by eGFRcr) with CVD.

Table S7. The relationship of eGFR categories with risk of incident CKD.

Table S8. Association of eGFR categories with risk of incident CVD stratified by age categories.

Table S9. Sensitivity analysis for the relationship of eGFR categories with risk of incident total cardiovascular disease.

Table S10. Disease definitions used in the UK Biobank study.

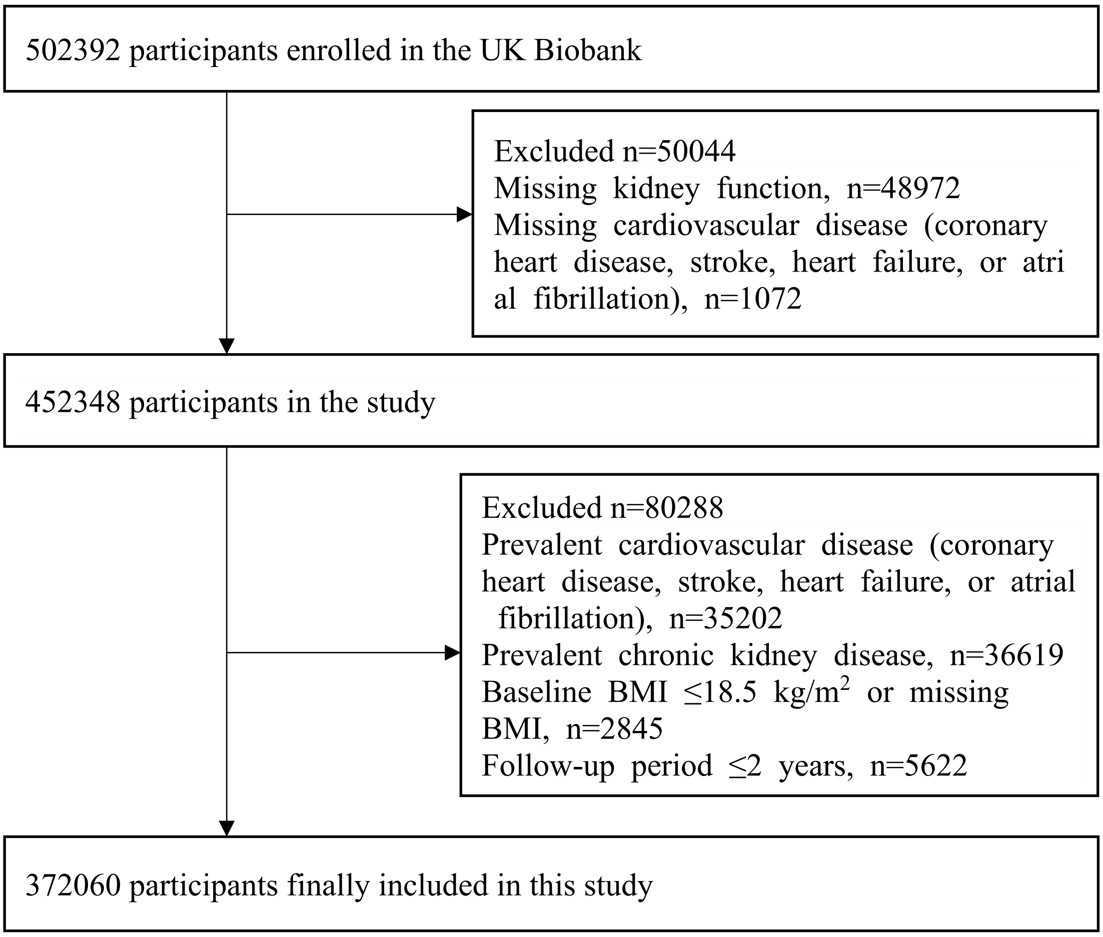

Figure S1. Flow chart of the participants in the current analysis.

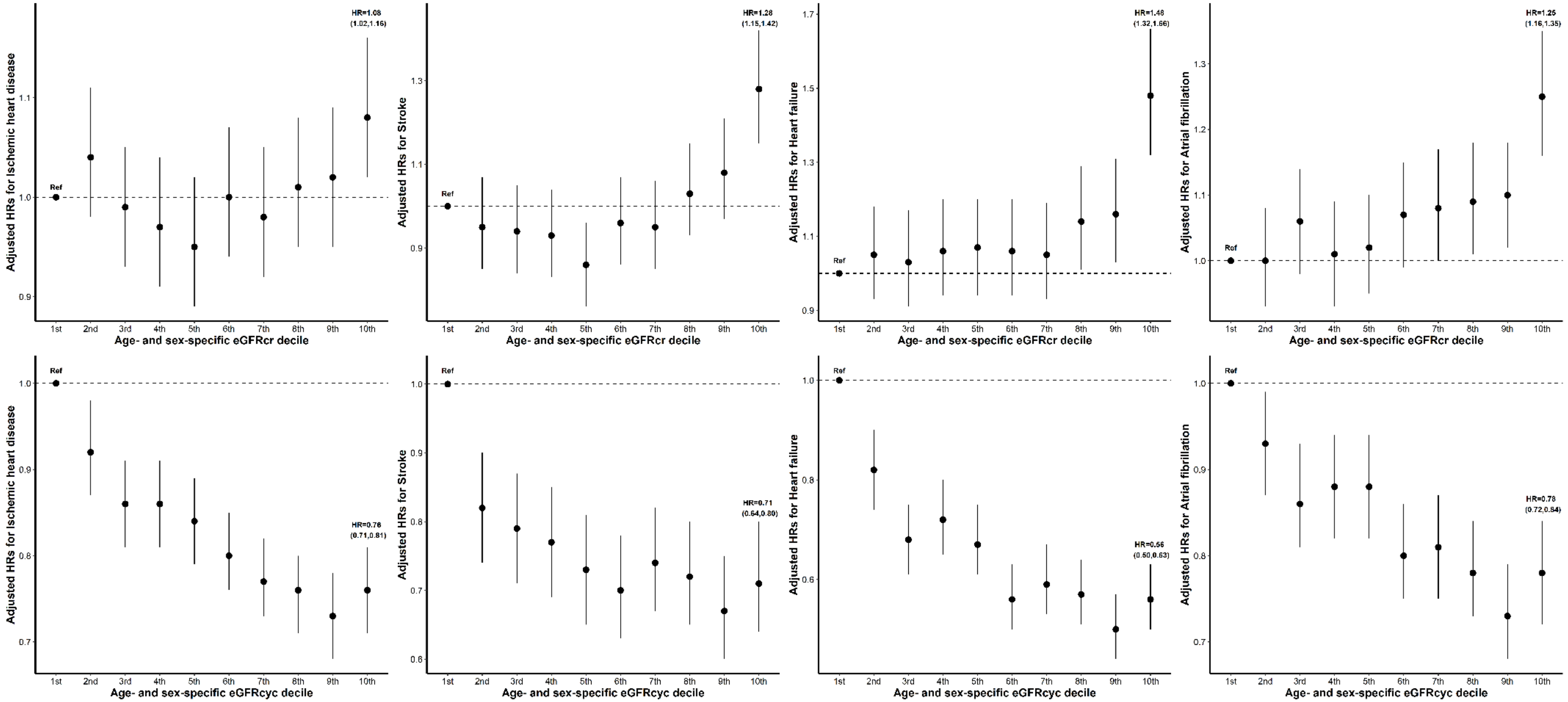

Figure S2. Adjusted hazard ratios for second cardiovascular outcomes by age- and sex-specific eGFR deciles.[*]

[*]Adjusted for age, sex, body mass index, race, systolic blood pressure, diastolic blood pressure, smoking status, history of diabetes, antihypertensive drug use, cholesterol-lowering drug use, triglycerides, total cholesterol, high-density lipoprotein cholesterol, and high-sensitivity

C reactive protein.

Abbreviation: eGFR, estimated glomerular filtration rate; eGFRcr, eGFR based on creatinine; eGFRcys, eGFR based on cystatin C.

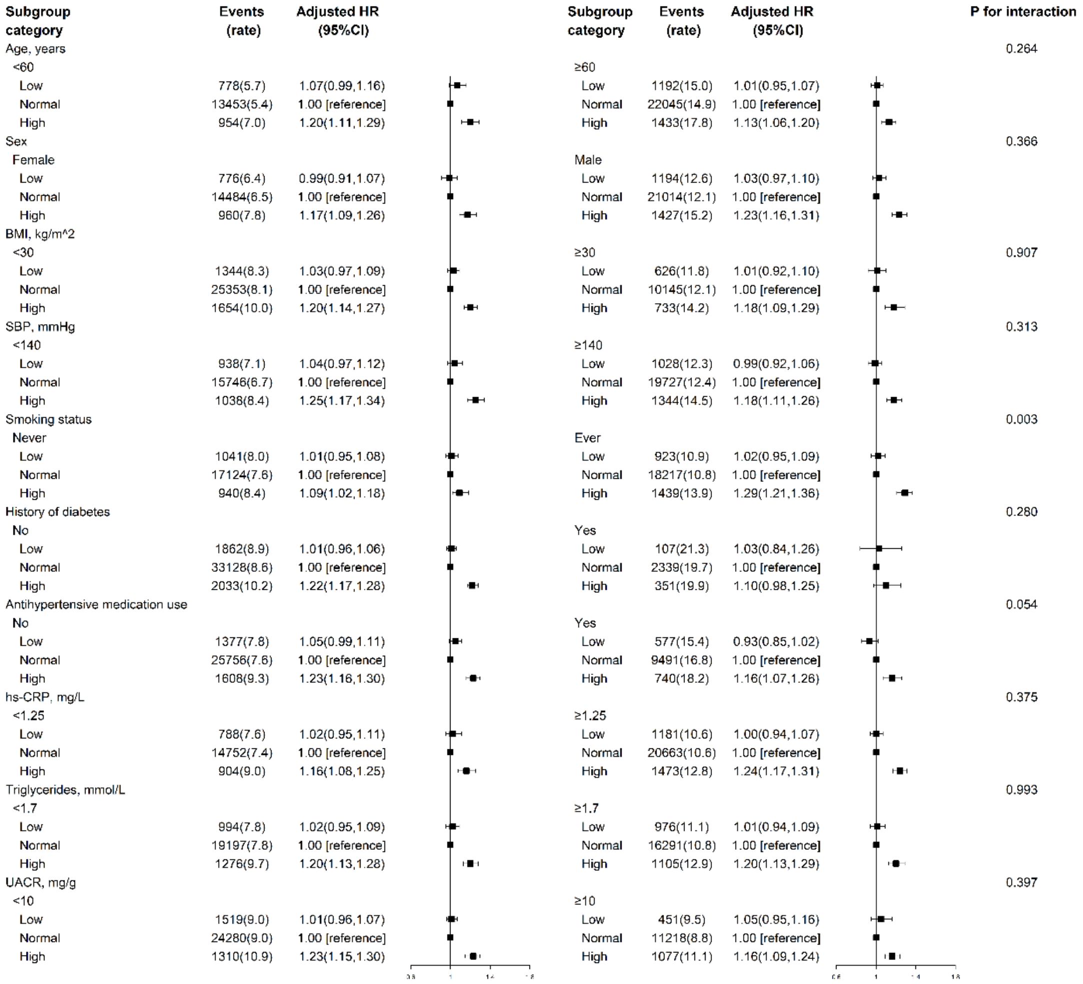

Figure S3. The association between eGFRcr categories and risk of incident CVD in various subgroups.*

* Adjusted for age, sex, body mass index, race, systolic blood pressure, diastolic blood pressure, smoking status, history of diabetes, antihypertensive drug use, cholesterol-lowering drug use, triglycerides, total cholesterol, high-density lipoprotein cholesterol, and high-sensitivity C reactive protein, if not already stratified.

Abbreviation: BMI, body mass index; CVD, cardiovascular disease; eGFR, estimated glomerular filtration rate; eGFRcr, eGFR based on creatinine; hs-CRP, high-sensitivity C reactive protein; SBP, systolic blood pressure; UACR, urine albumin: creatinine ratio.

Table S1. Distribution of eGFR by sex and age.*

Age, year	Female Low eGFR	Female Normal eGFR	Female High eGFR	Male Low eGFR	Male Normal eGFR	Male High eGFR
**eGFRcr, mL/min/1.73 m²**						
<45	<79.5	79.5-115.4	>115.4	<80.3	80.3-116	>116
45-50	<76.5	76.5-111.3	>111.3	<77.2	77.2-111.8	>111.8
50-55	<73.3	73.3-107.5	>107.5	<74.6	74.6-107.9	>107.9
55-60	<70.9	70.9-103.5	>103.5	<71.9	71.9-104.2	>104.2
60-65	<68.4	68.4-100	>100	<69.5	69.5-100.2	>100.2
≥65	<66.7	66.7-96.5	>96.5	<67.1	67.1-96.7	>96.7
**eGFRcyc, mL/min/1.73 m²**						
<45	<83.1	83.1-118.1	>118.1	<79.7	79.7-120.1	>120.1
45-50	<78.3	78.3-115.2	>115.2	<76.4	76.4-117	>117
50-55	<72.1	72.1-111	>111	<72.5	72.5-113.8	>113.8
55-60	<68.2	68.2-106.9	>106.9	<69.3	69.3-111	>111
60-65	<65.9	65.9-103.7	>103.7	<66.4	66.4-107.7	>107.7
≥65	<63.7	63.7-100.3	>100.3	<64	64-104.5	>104.5

* High eGFR was defined as an eGFR above the age- and sex-specific 95th percentile, and low eGFR was defined as an eGFR below the age- and sex-specific 5th percentile. Abbreviation: eGFR, estimated glomerular filtration rate; eGFRcr, eGFR based on creatinine; eGFRcys, eGFR based on cystatin C.

Table S2. Baseline population characteristics by the age- and sex-specific deciles of eGFRcr [*].

	Age- and sex-specific deciles of eGFRcr									
	**Decile 1**	**Decile 2**	**Decile 3**	**Decile 4**	**Decile 5**	**Decile 6**	**Decile 7**	**Decile 8**	**Decile 9**	**Decile 10**
**N**	37152	37139	37167	37230	37207	37222	37203	37197	37263	37280
**Age, years**	55.9 (8)	55.8 (8)	55.7 (8.1)	55.7 (8)	55.9 (8)	56.2 (8.1)	55.7 (8)	55.5 (8)	55.4 (8)	55.3 (8)
**Male, No. (%)**	16542 (44.5)	16538 (44.5)	16524 (44.5)	16570 (44.5)	16546 (44.5)	16592 (44.6)	16562 (44.5)	16549 (44.5)	16575 (44.5)	16579 (44.5)
**White, No. (%)**	35499 (95.6)	35602 (95.9)	35623 (95.8)	35612 (95.7)	35624 (95.7)	35752 (96.1)	35761 (96.1)	35596 (95.7)	35323 (94.8)	33018 (88.6)
**Body mass index, kg/m^2**	27.6 (4.3)	27.4 (4.3)	27.3 (4.3)	27.2 (4.4)	27.1 (4.5)	27.0 (4.4)	27.0 (4.5)	26.9 (4.6)	26.9 (4.7)	27.0 (5)
**Systolic blood pressure, mmHg**	136.6 (17.9)	136.7 (18)	136.8 (18)	137 (18.2)	137.3 (18.1)	137.4 (18.2)	137.2 (18.2)	137.2 (18.3)	137.4 (18.4)	137.9 (18.6)
**Diastolic blood pressure, mmHg**	82.2 (9.9)	82.3 (9.9)	82.3 (9.9)	82.4 (9.9)	82.4 (9.9)	82.2 (9.9)	82.2 (9.9)	82.1 (10)	82.2 (10)	82.3 (10.1)
**Smoking status, No. (%)**										
Never	22140 (59.6)	22027 (59.3)	21794 (58.6)	21584 (58)	21225 (57)	20927 (56.2)	20750 (55.8)	20341 (54.7)	20052 (53.8)	19175 (51.4)
Former	12342 (33.2)	12189 (32.8)	12344 (33.2)	12357 (33.2)	12439 (33.4)	12585 (33.8)	12554 (33.7)	12523 (33.7)	12525 (33.6)	12258 (32.9)
Current	2551 (6.9)	2819 (7.6)	2930 (7.9)	3173 (8.5)	3429 (9.2)	3595 (9.7)	3760 (10.1)	4234 (11.4)	4557 (12.2)	5717 (15.3)
**Antihypertensive drug use, No. (%)**	6456 (17.5)	5687 (15.4)	5531 (15.0)	5464 (14.8)	5541 (15.0)	5517 (14.9)	5405 (14.6)	5425 (14.7)	5535 (15.0)	6622 (17.9)
**Cholesterol-lowering drug use, No. (%)**	4836 (13.1)	4287 (11.6)	4267 (11.5)	4149 (11.2)	4139 (11.2)	4330 (11.7)	4104 (11.1)	4097 (11.1)	4175 (11.3)	4832 (13.1)
**History of diabetes, No. (%)**	1095 (3.1)	1026 (2.9)	1138 (3.2)	1127 (3.2)	1253 (3.5)	1450 (4.1)	1434 (4.1)	1576 (4.5)	1891 (5.3)	3086 (8.8)
**Total cholesterol, mmol/L**	5.7 (1.1)	5.8 (1.1)	5.8 (1.1)	5.8 (1.1)	5.8 (1.1)	5.8 (1.1)	5.8 (1.1)	5.8 (1.1)	5.8 (1.1)	5.7 (1.1)

**High-density lipoprotein cholesterol, mmol/L**	1.4 (0.4)	1.5 (0.4)	1.5 (0.4)	1.5 (0.4)	1.5 (0.4)	1.5 (0.4)	1.5 (0.4)	1.5 (0.4)	1.5 (0.4)	1.5 (0.4)
**Triglycerides, mmol/L**	1.7 (1)	1.7 (1)	1.7 (0.9)	1.7 (1)	1.7 (1)	1.7 (1)	1.7 (1)	1.7 (1)	1.7 (1.1)	1.8 (1.1)
**C-reactive protein, mg/L**	2.4 (3.8)	2.3 (3.8)	2.3 (3.6)	2.3 (3.8)	2.3 (4)	2.3 (3.8)	2.4 (4)	2.4 (3.9)	2.5 (4.2)	2.7 (4.5)
**Creatinine, mg/dL**	1 (0.1)	0.9 (0.1)	0.9 (0.1)	0.8 (0.1)	0.8 (0.1)	0.8 (0.1)	0.8 (0.1)	0.7 (0.1)	0.7 (0.1)	0.6 (0.1)
**Cystatin C, mg/L**	1 (0.1)	0.9 (0.1)	0.9 (0.1)	0.9 (0.1)	0.9 (0.1)	0.9 (0.1)	0.9 (0.1)	0.8 (0.1)	0.8 (0.1)	0.8 (0.1)
**eGFRcr, mL/min/1.73 m^2**	71.4 (5.7)	79.9 (5.2)	85.1 (5.4)	89.3 (5.6)	93.3 (5.7)	96.1 (5.6)	97.8 (5.7)	99.3 (5.7)	101.3 (5.9)	106 (7.2)
**eGFRcyc, mL/min/1.73 m^2**	83.1 (13.8)	86.2 (13.6)	88.2 (13.6)	89.7 (13.5)	91.1 (13.4)	92.4 (13.2)	94 (13.1)	95.5 (12.9)	97.4 (12.7)	100.3 (12.4)

*Values are presented as means (SD) or proportions.

Abbreviation: eGFR, estimated glomerular filtration rate; eGFRcr, eGFR based on creatinine; eGFRcys, eGFR based on cystatin C.

Table S3. Baseline population characteristics by the age- and sex-specific deciles of eGFRcyc *.

	Age- and sex-specific deciles of eGFRcyc									
	**Decile 1**	**Decile 2**	**Decile 3**	**Decile 4**	**Decile 5**	**Decile 6**	**Decile 7**	**Decile 8**	**Decile 9**	**Decile 10**
**N**	37176	37131	37214	37190	37203	37184	37207	37202	37281	37272
**Age, years**	55.9 (8)	55.9 (8)	55.8 (8.1)	55.8 (8)	55.7 (8)	55.7 (8)	55.7 (8)	55.7 (8)	55.6 (8.1)	55.4 (8)
**Male, No. (%)**	16538 (44.5)	16524 (44.5)	16549 (44.5)	16583 (44.6)	16541 (44.5)	16538 (44.5)	16573 (44.5)	16557 (44.5)	16567 (44.4)	16607 (44.6)
**White, No. (%)**	35092 (94.4)	35292 (95)	35427 (95.2)	35484 (95.4)	35526 (95.5)	35431 (95.3)	35387 (95.1)	35458 (95.3)	35330 (94.8)	34983 (93.9)
**Body mass index, kg/m^2**	29.8 (5.7)	28.6 (4.9)	27.9 (4.6)	27.4 (4.4)	27.1 (4.2)	26.8 (4.1)	26.4 (3.9)	26.1 (3.8)	25.8 (3.7)	25.3 (3.5)
**Systolic blood pressure, mmHg**	138.3 (18)	137.9 (18.3)	137.5 (18.1)	137.4 (18.1)	137.2 (18.1)	136.9 (18.2)	136.8 (18.1)	136.6 (18.3)	136.5 (18.2)	136.4 (18.5)
**Diastolic blood pressure, mmHg**	83.9 (10.1)	83.3 (10)	82.9 (10)	82.6 (9.9)	82.3 (9.8)	82 (9.8)	81.8 (9.8)	81.5 (9.9)	81.3 (9.8)	81 (9.8)
**Smoking status, No. (%)**										
Never	18794 (50.6)	20062 (54)	20549 (55.2)	20860 (56.1)	21317 (57.3)	21603 (58.1)	21756 (58.5)	21496 (57.8)	21817 (58.5)	21761 (58.4)
Former	11262 (30.3)	11705 (31.5)	12188 (32.8)	12248 (32.9)	12264 (33)	12483 (33.6)	12519 (33.6)	13049 (35.1)	13021 (34.9)	13377 (35.9)
Current	6971 (18.8)	5220 (14.1)	4353 (11.7)	3975 (10.7)	3497 (9.4)	2992 (8)	2829 (7.6)	2555 (6.9)	2336 (6.3)	2037 (5.5)
**Antihypertensive drug use, No. (%)**	8491 (23.1)	6895 (18.7)	6244 (16.9)	5750 (15.6)	5517 (14.9)	5194 (14)	4952 (13.4)	4875 (13.2)	4587 (12.4)	4678 (12.6)
**Cholesterol-lowering drug use, No. (%)**	5394 (14.6)	4592 (12.5)	4354 (11.8)	4245 (11.5)	4208 (11.4)	4049 (10.9)	4012 (10.8)	4113 (11.1)	3990 (10.8)	4259 (11.5)
**History of diabetes, No. (%)**	2170 (6.1)	1631 (4.6)	1476 (4.2)	1335 (3.8)	1332 (3.8)	1351 (3.8)	1274 (3.6)	1378 (3.9)	1400 (4)	1729 (4.9)
**Total cholesterol, mmol/L**	5.8 (1.1)	5.8 (1.1)	5.8 (1.1)	5.8 (1.1)	5.8 (1.1)	5.8 (1.1)	5.8 (1.1)	5.8 (1.1)	5.8 (1.1)	5.7 (1.1)

**High-density lipoprotein cholesterol, mmol/L**	1.3 (0.3)	1.4 (0.4)	1.4 (0.4)	1.4 (0.4)	1.5 (0.4)	1.5 (0.4)	1.5 (0.4)	1.5 (0.4)	1.6 (0.4)	1.6 (0.4)
**Triglycerides, mmol/L**	2 (1.1)	1.9 (1)	1.8 (1)	1.8 (1)	1.7 (1)	1.7 (1)	1.6 (1)	1.6 (0.9)	1.5 (0.9)	1.5 (0.9)
**C-reactive protein, mg/L**	3.8 (5.1)	3 (4.4)	2.7 (4.2)	2.5 (3.9)	2.3 (3.8)	2.2 (3.9)	2 (3.4)	1.9 (3.4)	1.8 (3.4)	1.6 (3.1)
**Creatinine, mg/dL**	0.9 (0.2)	0.8 (0.1)	0.8 (0.1)	0.8 (0.1)	0.8 (0.1)	0.8 (0.1)	0.8 (0.1)	0.8 (0.1)	0.7 (0.1)	0.7 (0.1)
**Cystatin C, mg/L**	1.1 (0.1)	1 (0.1)	0.9 (0.1)	0.9 (0.1)	0.9 (0.1)	0.9 (0)	0.8 (0)	0.8 (0)	0.8 (0)	0.7 (0.1)
**eGFRcr, mL/min/1.73 m^2**	84.7 (12.3)	87.3 (11.8)	89.1 (11.5)	90.4 (11.2)	91.5 (10.9)	92.7 (10.6)	93.8 (10.4)	95 (10.2)	96.4 (9.9)	98.8 (9.7)
**eGFRcyc, mL/min/1.73 m^2**	70 (6.7)	78 (7.3)	83 (7.8)	87 (8.2)	90.7 (8.3)	94.2 (8)	97.7 (7.6)	101.4 (7)	105.2 (6.3)	110.7 (6.8)

*Values are presented as means (SD) or proportions.

Abbreviation: eGFR, estimated glomerular filtration rate; eGFRcr, eGFR based on creatinine; eGFRcys, eGFR based on cystatin C.

Table S4. Baseline population characteristics by eGFRcr and eGFRcys categories *.

eGFRcys<br>eGFRcr	Low<br>Low	Low<br>Normal	Low<br>High	Normal<br>Low	Normal<br>Normal	Normal<br>High	High<br>Low	High<br>Normal	High<br>High
**N**	3296	15063	214	15115	305344	14473	164	14512	3879
**Age, years**	55.9 (7.8)	56 (8.1)	52.7 (8.2)	55.9 (8.1)	55.7 (8)	55.1 (8)	54.2 (8.2)	55.2 (8)	55.6 (8)
**Male, No. (%)**	1513 (45.9)	6657 (44.2)	96 (44.9)	6679 (44.2)	135981 (44.5)	6395 (44.2)	73 (44.5)	6411 (44.2)	1772 (45.7)
**White, No. (%)**	3131 (95)	14193 (94.2)	156 (72.9)	14425 (95.4)	292149 (95.7)	11999 (82.9)	153 (93.3)	13826 (95.3)	3378 (87.1)
**Body mass index, kg/m^2**	29.6 (5)	30.6 (6)	30.3 (8)	27.3 (4)	27 (4.3)	27.6 (5.5)	26 (3.9)	25.1 (3.4)	25.5 (3.9)
**Systolic blood pressure, mmHg**	137.7 (17.8)	138.6 (18.2)	135 (18.1)	136.4 (18)	137.1 (18.2)	138.4 (18.5)	133.4 (16.8)	136 (18.3)	138 (18.9)
**Diastolic blood pressure, mmHg**	84.1 (10.3)	84.2 (10.2)	81.9 (11)	82 (9.9)	82.2 (9.9)	82.8 (10.1)	80 (9.7)	80.7 (9.8)	81.5 (9.9)
**Smoking status, No. (%)**									
Never	1828 (55.5)	7190 (47.7)	92 (43)	9170 (60.7)	173675 (56.9)	7240 (50)	99 (60.4)	8593 (59.2)	2128 (54.9)
Former	1074 (32.6)	4400 (29.2)	56 (26.2)	5058 (33.5)	102327 (33.5)	4447 (30.7)	57 (34.8)	5273 (36.3)	1424 (36.7)
Current	383 (11.6)	3402 (22.6)	65 (30.4)	841 (5.6)	28408 (9.3)	2728 (18.8)	8 (4.9)	611 (4.2)	319 (8.2)
**Antihypertensive drug use, No. (%)**	892 (27.2)	3636 (24.4)	54 (25.7)	2514 (16.7)	44800 (14.8)	2881 (20.1)	13 (7.9)	1702 (11.8)	691 (18)
**Cholesterol-lowering drug use, No. (%)**	548 (16.7)	2265 (15.2)	23 (11)	1951 (13)	34235 (11.3)	2010 (14.1)	15 (9.1)	1617 (11.2)	552 (14.4)
**History of diabetes, No. (%)**	156 (5)	997 (7)	34 (16.7)	395 (2.8)	11082 (3.8)	1460 (10.7)	6 (4)	550 (4)	396 (10.8)
**Total cholesterol, mmol/L**	5.7 (1.1)	5.8 (1.2)	5.3 (1.5)	5.7 (1.1)	5.8 (1.1)	5.7 (1.2)	5.6 (1)	5.7 (1.1)	5.7 (1.2)

**High-density lipoprotein cholesterol, mmol/L**	1.3 (0.3)	1.3 (0.3)	1.2 (0.4)	1.5 (0.4)	1.5 (0.4)	1.4 (0.4)	1.6 (0.4)	1.6 (0.4)	1.6 (0.4)
**Triglycerides, mmol/L**	2 (1.1)	2.1 (1.1)	2.2 (1.4)	1.7 (1)	1.7 (1)	1.9 (1.2)	1.6 (1.1)	1.4 (0.9)	1.5 (1)
**C-reactive protein, mg/L**	3.3 (4.4)	4.3 (5.6)	5.3 (7.1)	2.2 (3.4)	2.3 (3.8)	3.1 (4.9)	1.9 (5.3)	1.5 (2.9)	2 (4)
**Creatinine, mg/dL**	1.1 (0.1)	0.9 (0.1)	0.6 (0.1)	1 (0.1)	0.8 (0.1)	0.6 (0.1)	1.1 (0.1)	0.7 (0.1)	0.6 (0.1)
**Cystatin C, mg/L**	1.1 (0.1)	1.1 (0.1)	1.1 (0.1)	0.9 (0.1)	0.9 (0.1)	0.8 (0.1)	0.7 (0.1)	0.7 (0.1)	0.7 (0.1)
**eGFRcr, mL/min/1.73 m^2**	67.7 (4.5)	86.8 (10.3)	111.5 (8.6)	68.2 (4.7)	92.4 (9.8)	108.2 (7.5)	68.4 (4.9)	97.8 (8.3)	108.3 (7.7)
**eGFRcyc, mL/min/1.73 m^2**	66.5 (5.1)	67 (5.4)	68.4 (5.9)	85.1 (12.3)	92 (12.5)	98.5 (11.1)	114.9 (8.5)	113 (7)	113.2 (6.8)

*Values are presented as means (SD) or proportions.

Abbreviation: eGFR, estimated glomerular filtration rate; eGFRcr, eGFR based on creatinine; eGFRcys, eGFR based on cystatin C.

Table S5. Combined association of eGFRcr categories and eGFRcyc categories with CVD.*

	Low eGFRcr		Normal eGFRcr		High eGFRcr	
	**Events (rate†)**	**HR (95%CI)**	**Events (rate†)**	**HR (95%CI)**	**Events (rate†)**	**HR (95%CI)**
**Low eGFRcyc**	426(11.5)	1.14(1.02, 1.26)	2426(14.1)	1.34(1.28, 1.40)	41(17.5)	1.88(1.33, 2.64)
**Normal eGFRcyc**	1526(8.6)	1.00(0.95, 1.06)	31862(8.8)	ref	1930(11.4)	1.26(1.20, 1.33)
**High eGFRcyc**	18(9.2)	1.35(0.81, 2.24)	1210(7.0)	0.90(0.85, 0.96)	416(9.1)	1.05(0.94, 1.17)

*Adjusted for age, sex, race, body mass index, systolic blood pressure, diastolic blood pressure, smoking status, history of diabetes, antihypertensive drug use, cholesterol-lowering drug use, triglycerides, total cholesterol, high-density lipoprotein cholesterol, and high-sensitivity C reactive protein.

† Incidence rates per 1000 person years.

Abbreviation: CVD, Cardiovascular disease; eGFR, estimated glomerular filtration rate; eGFRcr, eGFR based on creatinine; eGFRcys, eGFR based on cystatin C.

Table S6. Association of difference between eGFRcr and eGFRcys (eGFRcys minus eGFRcr divided by eGFRcr) with CVD.*

	Difference between eGFRcr and eGFRcys < -15% of eGFRcr		Difference between eGFRcr and eGFRcys within ±15% of eGFRcr		Difference between eGFRcr and eGFRcys > 15% of eGFRcr	
	**Events (rate†)**	**HR (95%CI)**	**Events (rate†)**	**HR (95%CI)**	**Events (rate†)**	**HR (95%CI)**
**Total**	3839(6.5)	0.88(0.84, 0.91)	27895(8.7)	ref	8121(14.0)	1.23(1.20, 1.27)
**Combined with eGFRcr categories**						
Low eGFRcr	2941(6.3)	0.86(0.82, 0.89)	25093(8.5)	ref	7464(13.8)	1.23(1.20, 1.27)
Normal eGFRcr	887(7.2)	0.99(0.92, 1.06)	1080(11.9)	1.09(1.02, 1.17)	3(7.3)	1.45(0.47, 4.49)
High eGFRcr	11(12.4)	1.24(0.67, 2.31)	1722(9.8)	1.16(1.10, 1.22)	654(15.9)	1.53(1.40, 1.67)
**Combined with eGFRcyc categories**						
Low eGFRcyc	3271(6.3)	0.89(0.86, 0.92)	25891(8.6)	ref	6156(13.8)	1.21(1.17, 1.25)
Normal eGFRcyc	3(3.8)	0.89(0.22, 3.56)	925(12.3)	1.24(1.15, 1.33)	1965(14.6)	1.38(1.32, 1.46)
High eGFRcyc	565(7.6)	0.83(0.76, 0.91)	1079(7.3)	1.00(0.94, 1.07)	0(0.0)	-

*Adjusted for age, sex, race, body mass index, systolic blood pressure, diastolic blood pressure, smoking status, history of diabetes, antihypertensive drug use, cholesterol-lowering drug use, triglycerides, total cholesterol, high-density lipoprotein cholesterol, and high-sensitivity C reactive protein.

† Incidence rates per 1000 person years.

Abbreviation: CVD, Cardiovascular disease; eGFR, estimated glomerular filtration rate; eGFRcr, eGFR based on creatinine; eGFRcys, eGFR based on cystatin C.

Table S7. The relationship of eGFR categories with risk of incident CKD.

CKD	eGFRcr			eGFRcyc		
	Low eGFR	Normal eGFR	High eGFR	Low eGFR	Normal eGFR	High eGFR
Events (Incidence rates*)	1406(6.3)	5084(1.2)	131(0.6)	1146(5.2)	5382(1.3)	93(0.4)
Crude model	5.20(4.90, 5.52)	ref	0.47(0.39, 0.56)	4.02(3.77, 4.28)	ref	0.31(0.25, 0.38)
Adjusted model 1†	5.10(4.78, 5.44)	ref	0.37(0.31, 0.45)	3.30(3.07, 3.54)	ref	0.32(0.26, 0.41)
Adjusted model 2†	5.06(4.74, 5.39)	ref	0.38(0.31, 0.46)	3.16(2.94, 3.40)	ref	0.33(0.27, 0.42)

*Incidence rates per 1000 person years.

†Adjusted Model 1: Adjusted for age, sex, race, systolic blood pressure, diastolic blood pressure, smoking status, history of diabetes, antihypertensive drug use, cholesterol-lowering drug use, triglycerides, total cholesterol, high-density lipoprotein cholesterol, and high-sensitivity C reactive protein.; Adjusted Model 2: adjusted for the covariates in Model 1 and further adjusted for body mass index.

Abbreviation: CKD, chronic kidney disease; eGFR, estimated glomerular filtration rate; eGFRcr, eGFR based on creatinine; eGFRcys, eGFR based on cystatin C.

Table S8. Association of eGFR categories with risk of incident CVD stratified by age categories.*

Age categories	Low eGFR		Normal eGFR		High eGFR		P for interaction
	Events (rate†)	HR (95%CI)	Events (rate†)	HR (95%CI)	Events (rate†)	HR (95%CI)	
**eGFRcr**							0.227
<45	67(2.5)	1.05(0.81,1.37)	1193(2.5)	ref	106(4.0)	1.50(1.20,1.88)	
45-50	134(4.1)	1.04(0.86,1.26)	2357(3.9)	ref	157(4.8)	1.18(0.99,1.40)	
50-55	222(6.2)	1.13(0.97,1.30)	3778(5.7)	ref	297(8.2)	1.29(1.13,1.47)	
55-60	355(8.8)	1.04(0.93,1.17)	6125(8.2)	ref	394(9.6)	1.09(0.97,1.22)	
60-65	594(12.3)	1.02(0.93,1.11)	10996(12.3)	ref	743(15.3)	1.19(1.09,1.29)	
≥65	598(19.0)	0.99(0.91,1.09)	11049(19.0)	ref	690(21.5)	1.09(1.00,1.19)	
**eGFRcyc**							0.006
<45	126(4.8)	1.56(1.28,1.91)	1193(2.5)	ref	47(1.8)	0.69(0.50,0.97)	
45-50	232(7.2)	1.47(1.27,1.70)	2316(3.9)	ref	100(3.0)	0.82(0.65,1.02)	
50-55	376(10.6)	1.54(1.37,1.72)	3760(5.7)	ref	161(4.3)	0.80(0.67,0.95)	
55-60	517(13.1)	1.32(1.20,1.46)	6075(8.2)	ref	282(6.8)	0.92(0.81,1.04)	
60-65	863(18.4)	1.28(1.18,1.37)	10956(12.3)	ref	514(10.2)	0.93(0.84,1.02)	
≥65	779(25.4)	1.21(1.12,1.31)	11018(18.9)	ref	540(16.5)	0.93(0.85,1.02)	

*Adjusted for sex, race, body mass index, systolic blood pressure, diastolic blood pressure, smoking status, history of diabetes, antihypertensive drug use, cholesterol-lowering drug use, triglycerides, total cholesterol, high-density lipoprotein cholesterol, and high-sensitivity C reactive protein.

† Incidence rates per 1000 person years.

Abbreviation: CVD, Cardiovascular disease; eGFR, estimated glomerular filtration rate; eGFRcr, eGFR based on creatinine; eGFRcys, eGFR based on cystatin C.

Table S9. Sensitivity analysis for the relationship of eGFR categories with risk of incident total cardiovascular disease.*

eGFR, mL/min/1.73 m^2	eGFRcr		eGFRcyc	
	No of events	HR (95%CI)*	No of events	HR (95%CI)*
Sensitivity analysis 1: Using different methods to define eGFR categories				
**Per SD increment**	39855	1.03(1.02, 1.05)	39855	0.89(0.88, 0.90)
**Quartiles**				
Q1	11927	ref	15407	ref
Q2	11800	0.99(0.96, 1.01)	10867	0.88(0.86, 0.91)
Q3	10100	1.04(1.01, 1.07)	8279	0.83(0.81, 0.86)
Q4	6028	1.15(1.11, 1.20)	5302	0.76(0.74, 0.79)
**P for trend**		<0.001		<0.001
**Clinical categories**				
≥120	70	1.36(1.04, 1.78)	132	0.90(0.74, 1.08)
105-<120	2463	1.16(1.10, 1.22)	3995	0.91(0.88, 0.95)
90-<105	18954	ref	11426	ref
75-<90	13559	0.95(0.93, 0.98)	14840	1.09(1.07, 1.12)
60-<75	4809	0.99(0.96, 1.03)	9462	1.26(1.22, 1.30)
Sensitivity analysis 2: Using Cox model stratified by age and sex				
**Low**	1970	1.01(0.97, 1.07)	2893	1.29(1.24, 1.35)
**Normal**	35498	ref	35318	ref
**High**	2387	1.21(1.15, 1.26)	1644	0.92(0.88, 0.98)
Sensitivity analysis 3: Using Cox model without adjustment for age and sex				
**Low**	1970	1.04(0.99, 1.09)	2893	1.26(1.21, 1.32)
**Normal**	35498	ref	35318	ref
**High**	2387	1.12(1.07, 1.17)	1644	0.90(0.86, 0.95)
Sensitivity analysis 4: Using Fine-Gray competing risk model				
**Low**	1970	1.01(0.96,1.06)	2893	1.27(1.21,1.32)
**Normal**	35498	ref	35318	ref
**High**	2387	1.20(1.14,1.25)	1644	0.93(0.88,0.98)

***** Adjusted for age, sex, race, body mass index, systolic blood pressure, diastolic blood pressure, smoking status, history of diabetes, antihypertensive drug use, cholesterol-lowering drug use, triglycerides, total cholesterol, high-density lipoprotein cholesterol, and high-sensitivity C reactive protein.

Abbreviation: eGFR, estimated glomerular filtration rate; eGFRcr, eGFR based on creatinine; eGFRcys, eGFR based on cystatin C.

Table S10. Disease definitions used in the UK Biobank study.

Disease	**ICD-9**	**ICD-10**	**OPCS-4**
Ischemic heart disease	410–414	I20-I25	K40-K46, K49, K50, K75
Stroke	430, 431, 434, 436	I60, I61, I63, I64, I629, I678, I690, I693	A05.2-A05.4, L35.1, L35.3, L34.3
Atrial Fibrillation	4273	I48	K62.1-K62.4
Heart failure	428	I11.0, I13.0, I13.2, I50.0, I50.1, I50.9	-
Chronic kidney disease	58	I12.0, I13.1, I13.2, N18.0, N18.3, N18.4, N18.5, N18.8, N18.9	M01

Abbreviations: ICD, International Classification of Diseases; OPCS, the Office of Population Censuses and Surveys Classification of Interventions and Procedures.
